# Trajectories of symptom scores and risk of cardiovascular events in the vulnerable phase of heart failure: an analysis using a latent class trajectory model

**DOI:** 10.3389/fcvm.2026.1841870

**Published:** 2026-07-20

**Authors:** Dan Xiao, Yuan Xu, Yan Jiang, Zhiteng Xiong, Qihui Shen, Xinglan Sun, Xiaoyun Xiong, Bing Hu

**Affiliations:** 1 Department of Nursing, The Second Affiliated Hospital of Nanchang University, Nanchang, Jiangxi, China; 2 School of Nursing, Jiangxi Medical College, Nanchang University, Nanchang, Jiangxi, China; 3 Department of Acupuncture and Tuina, the Affiliated Hospital of Jiangxi University of Chinese Medicine, Nanchang, Jiangxi, China

**Keywords:** cardiovascular events, heart failure, latent class trajectory model, trajectories, vulnerable phase

## Abstract

**Background:**

The first 2–3 months after discharge for acute or decompensated chronic heart failure (HF) constitute a “vulnerable phase” marked by high risks of rehospitalization and death. Symptom fluctuations during this period may be associated with adverse outcomes, yet evidence on post-discharge symptom trajectories is limited. This study applied a latent class trajectory model to characterize KCCQ symptom evolution and its association with early cardiovascular events.

**Methods:**

In this prospective cohort study, 1,109 patients with chronic HF admitted to a tertiary hospital in Nanchang, China (August 2020–July 2025), completed the Kansas City Cardiomyopathy Questionnaire (KCCQ) at six time points: day 3 after admission, at discharge, and 1, 2, 3, and 6 months post-discharge. Symptom trajectories were identified using latent class trajectory modeling. Associations between trajectory groups and major adverse cardiovascular events (MACEs) were analyzed using multivariable logistic regression, and an exploratory mediation analysis was conducted to examine whether trajectory patterns partly accounted for the association between KCCQ scores and MACEs.

**Results:**

During follow-up, 207 patients (18.7%) experienced MACEs. Latent class trajectory analysis identified four distinct groups: persistently declining, slowly improving, stable-moderate, and stable-high. Relative to the stable-moderate group, patients in the persistently declining group had higher odds of MACEs (OR = 2.242, 95% CI: 1.073–4.718, *P* = 0.032). The exploratory mediation analysis suggested a statistically significant indirect association through trajectory patterns (*β*=–0.007, *P* < 0.001).

**Conclusions:**

Symptom recovery after HF hospitalization is heterogeneous and was associated with cardiovascular outcomes. Persistently worsening symptoms predict the highest risk, while stable or improving trajectories indicate favorable recovery. Tracking KCCQ trajectories may support early risk stratification and personalized management during the vulnerable phase.

## Introduction

Heart failure (HF) represents the severe and terminal stage of various cardiac diseases. With population aging and increasing prevalence of cardiovascular disease, HF has become a major public health concern in the 21st century (1, 2). The first 2–3 months after discharge in patients with acute or decompensated chronic HF constitute a “vulnerable phase,” during which mortality and rehospitalization rates reach approximately 15% and 30%, respectively (3, 4).

Elevated risk during this phase is often associated with hemodynamic deterioration, short hospital stays, poor adherence, unresolved congestion at discharge, or incomplete evaluation of underlying causes (5, 6). Without adequate management, patients are prone to recurrent decompensation, repeated hospitalization, and rapid progression to end-stage HF or death; appropriate intervention can facilitate a smooth transition to chronic HF.

Notably, not all high-risk patients exhibit overt symptoms. Some present only with mild or intermittent “subthreshold symptoms,” which may be overlooked but still predict rehospitalization and cardiovascular events (7). Previous studies have shown that patient-reported symptoms and health status are closely associated with subsequent hospitalization and mortality in patients with HF (8). However, symptoms after discharge are not static; they may improve, remain stable, fluctuate, or deteriorate over time. Therefore, a single cross-sectional assessment may be insufficient to capture the dynamic clinical risk during the vulnerable phase. Longitudinal follow-up allows dynamic monitoring of symptom changes, and latent class trajectory modeling can identify distinct evolution patterns, improving risk stratification and supporting individualized management.

Although previous research has established the clinical importance of the vulnerable phase after HF hospitalization and has explored the prognostic value of repeated symptom or health-status assessments (9), evidence on symptom trajectories during this early post-discharge period remains relatively limited, particularly in Chinese prospective cohorts (10). Building on this prior evidence, the present study used longitudinal symptom assessment data and latent class trajectory modeling to identify distinct symptom trajectory patterns during the vulnerable phase and to evaluate their associations with early cardiovascular events. This study aimed to provide additional cohort-specific evidence to support risk stratification and individualized post-discharge management in patients with HF.

## Methods

### Study design and population

This prospective cohort study consecutively enrolled patients with chronic heart failure (HF) admitted to a Class III Grade A hospital in Nanchang, Jiangxi Province, China, between August 2020 and July 2025. A total of 1,109 patients were included based on the following criteria: Inclusion criteria: (1) diagnosis of acute exacerbation of chronic HF according to the 2016 ESC Heart Failure Guidelines (11); (2) absence of psychiatric disorders or cognitive impairment, with the ability to report symptoms and experiences; (3) good adherence and willingness to participate in follow-up; Exclusion criteria: (1) acute coronary syndrome; (2) refractory end-stage HF; (3) incomplete clinical data; (4) severe comorbidities in other organ systems; Elimination criteria: (1) death or impaired consciousness preventing survey completion; (2) voluntary withdrawal from treatment with an expected survival of <3 months.

### Data collection

In accordance with the clinical management characteristics of acute exacerbations of chronic heart failure, eligible patients were assessed at six predefined time points: the third day after admission (T0), the day of discharge (T1), and at 1 month (T2), 2 months (T3), 3 months (T4), and 6 months (T5) following discharge. In this study, the vulnerable phase was defined primarily as the early post-discharge period after hospitalization for acute exacerbation of chronic HF. The day-3 in-hospital assessment was included to capture patients' early stabilization status during hospitalization and to provide a pre-discharge baseline reference for subsequent post-discharge trajectory analysis. Baseline information was extracted from the hospital electronic medical record system, and all laboratory parameters were measured by the hospital's central laboratory.

### Data measurement

#### Baseline characteristics of the patients

The survey instrument was developed by the investigators and included three major components. The first component collected demographic information, such as age, sex, and educational level. The second component captured laboratory and clinical characteristics, including heart failure–related parameters [disease duration, number and length of hospitalizations, presence of peripheral edema during hospitalization, New York Heart Association (NYHA) functional class, and left ventricular ejection fraction], comorbidities [hypertension, diabetes mellitus, stroke, coronary artery disease, and atrial fibrillation], and laboratory results [B-type natriuretic peptide (BNP) at admission, percentage reduction in BNP during hospitalization, BNP at discharge, glycated hemoglobin, serum urea at discharge, and serum sodium at both admission and discharge]. The third component assessed follow-up data on vital signs and clinical outcomes, including heart rate at discharge and at 3 months post-discharge, systolic blood pressure during hospitalization, diastolic blood pressure at admission, systolic and diastolic blood pressure at discharge, blood pressure at 2 and 4 weeks after discharge, as well as the occurrence of major adverse cardiovascular events during follow-up.

### Kansas city cardiomyopathy questionnaire

Heart failure–related quality of life was assessed using the Chinese version of the Kansas City Cardiomyopathy Questionnaire (KCCQ), translated and validated by Deng et al. (12). The instrument contains 23 items covering eight domains, including physical function, symptom frequency and severity, social function, and quality of life. Scores range from 0 to 100, with lower scores indicating greater impairment due to heart failure. Except for the self-perception domain, Cronbach's α coefficients for all domains exceeded 0.8, indicating good reliability and validity of the scale (12).

### Self-care of heart failure inventory

Self-care ability was measured using the Chinese version of the Self-Care of Heart Failure Inventory (SCHFI), translated and validated by Chen et al. (13). The inventory consists of 22 items across three dimensions: self-care maintenance (10 items), self-care management (6 items), and self-care confidence (6 items). Most items are scored on a 4-point scale, except for two items in the management subscale, which use a 5-point scale. Higher scores reflect better self-care ability. The overall Cronbach's α coefficient of the instrument was 0.853, demonstrating good reliability and validity (13).

### Definition of major adverse cardiovascular events

The primary outcome was the occurrence of major adverse cardiovascular events (MACEs) within the predefined 6-month follow-up period. MACEs were defined as a composite outcome including heart failure–related rehospitalization, heart failure–related death, atrial fibrillation, malignant arrhythmia, cardiorenal syndrome, and other clinically documented adverse cardiovascular events.

### Quality control

Researchers explained the purpose and significance of the study to eligible participants or their family members and emphasized their right to participate or decline without consequence. Standardized terminology, variable definitions, and uniform interviewing and testing procedures were applied. Questionnaires were administered directly by the investigators at all six time points. For patients returning for follow-up, face-to-face surveys were conducted; for those unable to return, follow-up surveys were completed via telephone or online platforms according to participants' preference. Investigators addressed all participant questions during the process. Completed questionnaires were reviewed by two independent researchers, and responses showing obvious patterns or duplication were excluded.

### Trajectory modeling

Group-based trajectory modeling (GBTM) was employed to identify heterogeneous longitudinal patterns of Kansas City Cardiomyopathy Questionnaire (KCCQ) total scores among patients with chronic heart failure during follow-up. The analysis was performed using the trajeR package in R (Version 0.11.1), based on KCCQ assessments collected at six prespecified time points, to capture dynamic changes in health status over time. To determine the optimal trajectory structure, models specifying two to five trajectory groups were fitted and compared. Within the candidate trajectory models, polynomial functions were used to model longitudinal changes in KCCQ scores, and different polynomial specifications were further evaluated to account for the potential nonlinearity of symptom evolution in heart failure. Model selection was guided by a combination of Bayesian information criterion (BIC), Akaike information criterion (AIC), entropy, goodness of fit, and parsimony. The final model was chosen according to the following criteria: (1) relatively optimal BIC and AIC values; (2) higher entropy, indicating better classification certainty; (3) each trajectory group comprising more than 5% of the study population; and (4) average posterior probability of group assignment ≥70%, indicating adequate classification accuracy. To evaluate model robustness, sensitivity analyses were conducted by comparing alternative group solutions and different polynomial specifications, including linear, quadratic, and cubic trajectory forms. Average trajectories for each latent class were plotted, and clinical labels were assigned based on trajectory patterns. Baseline demographic and clinical characteristics were then compared across trajectory groups.

### Mediation analysis

To explore whether the effect of baseline KCCQ on cardiovascular events was mediated through the KCCQ trajectory groups, mediation analysis was conducted using the mediation package (version 4.5.1) in R software (version 4.2.3). The analysis was performed to assess the plausibility of the causal hypothesis linking KCCQ trajectories and cardiovascular events. Indirect effects (i.e., the a × b path) were estimated based on regression models, and their significance was tested using the nonparametric percentile bootstrap method. Specifically, the following steps were conducted: (1) the effect of baseline KCCQ on the mediator (KCCQ trajectory groups) was tested (a path); (2) the effect of KCCQ trajectory groups on cardiovascular events was examined while controlling for baseline KCCQ (b path); and (3) the direct effect of baseline KCCQ on cardiovascular events was assessed (c path). The indirect effect was calculated as the product of coefficients a and b, with 95% confidence intervals obtained by bootstrapping with 500 resamples.

### Statistical analysis

Statistical analyses were performed using R software (version 4.2.3) and Python (version 3.11.4). Missing data were imputed using multiple imputation by chained equations with the mice package in R software. Continuous variables were expressed as mean ± standard deviation or median (interquartile range), and categorical variables as frequencies and percentages. Between-group comparisons were conducted using one-way analysis of variance, Kruskal–Wallis test, or chi-square test, as appropriate. The primary outcome was defined as the occurrence of MACEs within a predefined 6-month follow-up window. Multivariable logistic regression was used to estimate the association between symptom trajectory groups and MACE occurrence within the predefined 6-month follow-up period, with adjustment for potential confounders. Model 1 adjusted for sex, age, and educational level; Model 2 was further expanded to include key demographic and clinical variables, including age, sex, educational level, NYHA functional class, diabetes mellitus, stroke, coronary artery disease, atrial fibrillation, serum sodium at discharge, number of HF hospitalizations, BNP at discharge, heart rate at discharge, and serum urea at discharge. Subgroup analyses were performed according to hypertension status and peripheral edema. All tests were two-tailed, and *P* < 0.05 was considered statistically significant.

## Result

### Participants

**Figure 1:**
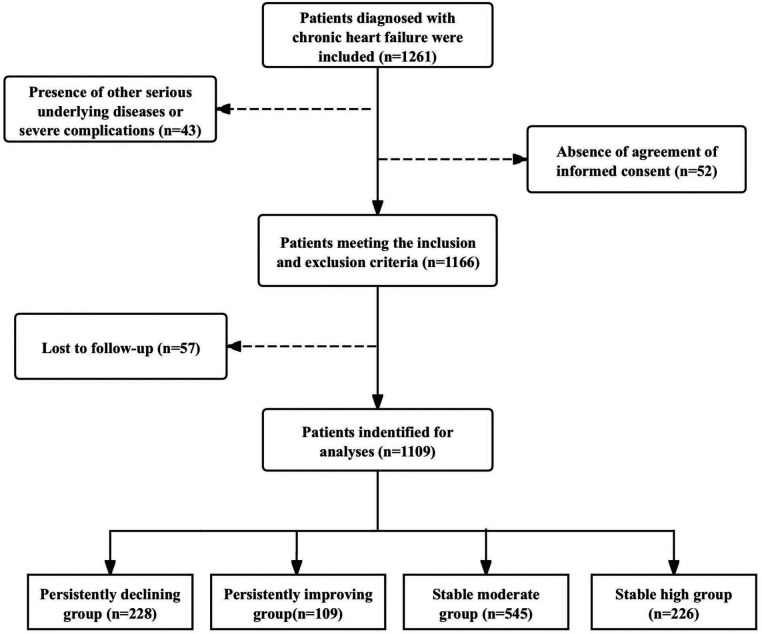
Patient screening and enrollment flowchart.

A total of 1,261 patients with chronic heart failure were initially assessed. After excluding 95 patients due to severe comorbidities or lack of informed consent, 1,166 patients met the inclusion criteria. During follow-up, 57 patients were lost to follow up, resulting in a final sample of 1,109 patients for analysis. The patient selection flowchart is shown in Figure 1.

### Cohort characteristics

Among the 1,109 enrolled patients, 207 (18.7%) experienced MACEs during follow-up. Compared with patients without MACEs, those with MACEs were more likely to be female (47.34% vs. 39.13%, *P* = 0.037), had more prior heart failure hospitalizations [13 vs. 10, *P* < 0.001], and had a higher proportion of NYHA class IV (24.64% vs. 22.17%, *P* = 0.023). They also had higher heart rates at discharge and at 3 months post-discharge, as well as a higher incidence of peripheral edema during hospitalization (43.00% vs. 30.04%, *P* < 0.001). Laboratory findings showed higher BNP levels at admission and discharge, a smaller percentage reduction in BNP during hospitalization (43.03% vs. 46.95%, *P* = 0.010), lower serum sodium (135.93 vs. 138.05 mmol/L, *P* < 0.001), and higher serum urea (10.67 vs. 9.76 mmol/L, *P* < 0.001) in the MACEs group. No significant differences were observed in age, educational level, or baseline KCCQ total score. Full baseline characteristics are presented in Table 1.

**Table 1:** Baseline characteristics in patients with heart failure.

Variable	Overall	Maces	No Maces	*P*
	(*N* = 1,109)	(*N* = 207)	(*N* = 907)	
Demographic and clinical characteristics				
Age, M (SD)	67.90 ± 14.85	67.81 ± 14.71	67.92 ± 14.88	0.920
Gender, *n* (%)				
Male	658 (59.33%)	109 (52.66%)	549 (60.87%)	0.037
Female	451 (40.67%)	98 (47.34%)	353 (39.13%)	
Educational level, *n* (%)				
Primary school and below	441 (39.77%)	360 (39.91%)	81 (39.13%)	0.563
Middle School	148 (13.35%)	126 (13.97%)	22 (10.63%)	
High school/vocational school	344 (31.02%)	275 (30.49%)	69 (33.33%)	
University and above	176 (15.87%)	141 (15.63%)	35 (16.91%)	
Number of hospitalizations due to heart failure, MED [IQR]	12.00 [8.00, 18.00]	13.00 [9.00, 20.50]	10.00 [8.00, 16.00]	<0.001
Cardiac function classification, *n* (%)				
Level 1	44 (3.97%)	1 (0.48%)	43 (4.77%)	0.023
Level 2	336 (30.30%)	58 (28.02%)	278 (30.82%)	
Level 3	478 (43.10%)	97 (46.86%)	381 (42.24%)	
Level 4	251 (22.63%)	51 (24.64%)	200 (22.17%)	
HR at discharge, M (SD)	72.63 ± 13.57	75.55 ± 13.32	71.96 ± 13.54	<0.001
Peripheral edema during hospitalization, *n* (%)	360 (32.46%)	89 (43.00%)	271 (30.04%)	<0.001
SBP at 2 weeks post-discharge, mmHg, M (SD)	122.79 ± 17.41	116.70 ± 16.80	124.19 ± 17.25	<0.001
DBP at 2 weeks post-discharge, mmHg, M (SD)	70.17 ± 12.64	67.68 ± 13.34	70.75 ± 12.40	0.002
SBP at 4 weeks post-discharge, mmHg, M (SD)	117.62 ± 17.51	113.14 ± 17.16	118.65 ± 17.43	<0.001
DBP at 4 weeks post-discharge, mmHg, M (SD)	76.45 ± 13.95	74.22 ± 13.32	76.97 ± 14.04	0.011
HR at 3 months post-discharge, MED [IQR]	78.00 [67.00, 87.00]	89.000 [88.00,95.00]	76.000 [65.00, 82.00]	<0.001
Baseline KCCQ score, M (SD)	84.21 ± 9.08	85.07 ± 7.88	84.01 ± 9.33	0.095
Complication				
Hypertension, *n* (%)	805 (72.59%)	646 (71.62%)	159 (76.81%)	0.154
Coronary heart disease, *n* (%)	81 (7.30%)	25 (12.08%)	56 (6.21%)	0.005
Diabetes, *n* (%)	173 (15.60%)	44 (21.26%)	129 (14.30%)	0.054
Stroke, *n* (%)	63 (5.68%)	54 (5.99%)	9 (4.35%)	0.452
Atrial fibrillation, *n* (%)	77 (6.94%)	58 (6.43%)	19 (9.18%)	0.211
Chemistry				
BNP at admission, M (SD)	2,707.36 ± 2,558.64	3,100.03 ± 2,923.71	2,617.25 ± 2,458.42	0.014
Percentage decrease in BNP during hospitalization, M (SD)	46.22 ± 19.66	43.03 ± 20.76	46.95 ± 19.33	0.010
BNP at discharge, M (SD)	961.70 ± 1,071.48	1,123.06 ± 853.23	924.67 ± 1,112.25	0.016
Serum sodium at discharge, M (SD)	137.65 ± 6.02	135.93 ± 6.71	138.05 ± 5.77	<0.001
Serum urea at discharge, M (SD)	9.93 ± 3.15	10.67 ± 3.45	9.76 ± 3.05	<0.001

HR, heart rate; SBP, systolic blood pressure; DBP, diastolic blood pressure; KCCQ, kansas city cardiomyopathy questionnaire; BNP, B-type natriuretic peptide.

### Trajectory modeling results

Using GBTM, models with two to five latent trajectory groups were fitted and compared based on AIC, BIC, entropy, group size, classification accuracy, and clinical interpretability. As shown in Table 2, the four-group model had the lowest BIC and AIC values among the candidate models (BIC = 39,645.12, AIC = 39,549.91), indicating the best balance between model fit and parsimony. Although the five-group model generated an additional latent class, it did not improve model fit, as reflected by higher BIC and AIC values. Therefore, the four-group solution was selected as the optimal latent class structure, with detailed model fit indices and classification diagnostics presented in Supplementary Tables S1.

**Table 2:** Model fit comparisons and latent class prevalence by model.

NG	BIC	AIC	Entropy
2	44,507.82	44,462.72	1
3	41,841.34	41,771.18	1.0036
4	39,645.12	39,549.91	1.0048
5	39,680.18	39,559.91	1.0041

AIC, Akaike information criterion; BIC, Bayesian information criterion;·Min AvePP, minimum average posterior probability; categorical probability, estimated class membership probability (%).

To further examine model robustness and address the possibility of nonlinear symptom evolution in heart failure, sensitivity analyses were conducted by comparing linear, quadratic, and cubic polynomial specifications within the four-group model. The quadratic model showed the lowest BIC and AIC values (BIC = 39,645.12, AIC = 39,549.91), supporting its superior model fit compared with the linear and cubic specifications. Therefore, the final model was specified as a four-group quadratic trajectory model.

**Figure 2:**
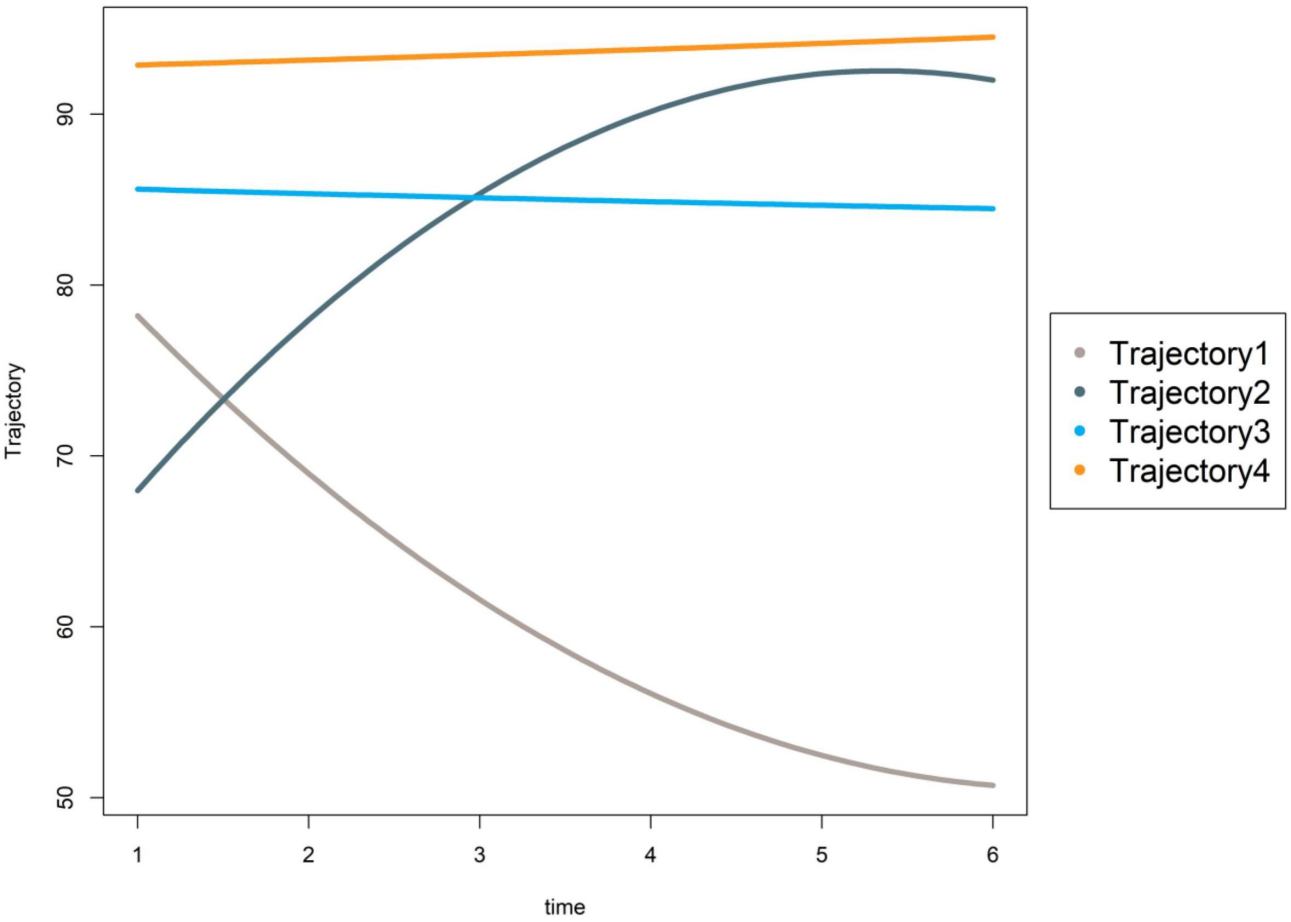
Mean and individual KCCQ overall summary score trajectories for the 4 trajectory groups.

In the final model, the proportions of Groups 1–4 were 20.6%, 9.8%, 49.2%, and 20.4%, respectively. The average posterior probabilities for group assignment were 1.000, 0.998, 0.996, and 0.998, respectively, all exceeding the commonly recommended threshold of 0.70. These findings indicated excellent classification accuracy and supported the reliability of the final trajectory classification, as shown in Figure 2 and Supplementary Table S2.

### Heart failure symptom trajectories

**Figure 3:**
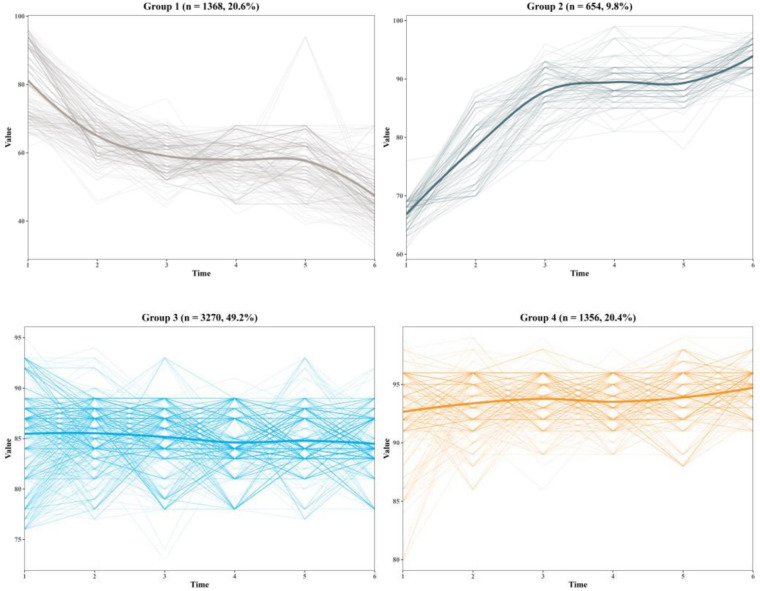
Distinct symptom evolution patterns among four phenotypic subgroups in heart failure.

Based on longitudinal follow-up data, the latent class trajectory model categorized patients into four distinct HF symptom trajectory groups (Figure 3). The characteristics of each trajectory group were as follows: Group 1 (Persistently declining group, *n* = 228, 20.6%): Patients in this group exhibited a clear worsening of HF symptoms over time, indicating potential disease progression and an increasing symptom burden during follow-up; Group 2 (Persistently improving group, *n* = 109, 9.8%): Patients showed a gradual improvement in symptoms, suggesting effective therapeutic interventions or enhanced self-management, with a progressive reduction in symptom burden; Group 3 (Stable moderate group, *n* = 545, 49.2%): Patients maintained relatively stable symptom scores at a moderate level, reflecting a steady, intermediate symptom burden over time; Group 4 (Stable high group, *n* = 226, 20.4%): Patients consistently exhibited high scores, indicative of minimal or near-absent symptoms. Overall, most patients maintained stable symptoms throughout follow-up, with only a minority experiencing marked deterioration or improvement. These findings highlight the substantial heterogeneity in HF symptom evolution and underscore the need for stratified clinical management based on trajectory characteristics to optimize treatment outcomes and resource allocation. The distribution of MACEs differed across the four trajectory groups. MACEs occurred in 80 of 228 patients in the persistently declining group (35.1%), 9 of 109 patients in the gradually improving group (8.2%), 106 of 545 patients in the Stable moderate group (19.4%), and 12 of 226 patients in the stable high-score group (5.3%).

### Clinical characteristics of patients according to KCCQ symptom scores

A total of 1,109 HF patients were included and categorized into four groups based on KCCQ symptom scores. Significant differences among the four groups were observed in educational level, NYHA functional class, heart rate, blood pressure, comorbidities (hypertension, stroke, coronary heart disease, diabetes, and atrial fibrillation), previous hospitalizations, peripheral edema, BNP levels, serum sodium, and serum urea. Specifically, Group 1 exhibited the poorest cardiac function, the lowest overall educational level, the highest number of prior hospitalizations, the greatest prevalence of peripheral edema, and markedly elevated BNP levels. Group 2 showed relatively lower blood pressure and heart rate at discharge and the greatest reduction in BNP levels. Group 3, the largest group, generally displayed intermediate clinical and laboratory characteristics. Group 4 demonstrated the best cardiac function, the fewest prior hospitalizations, the lowest BNP and serum urea levels, and the highest serum sodium concentration. Regarding comorbidities, Group 1 had the highest prevalence of hypertension, diabetes, coronary heart disease, stroke, and atrial fibrillation, whereas Group 4 carried the lowest comorbidity burden. Detailed results are presented in Table 3.

**Table 3:** Characteristics of study patients (*N* = 1,109).

Variables	Overall (*n* = 1,109)	Kansas city cardiomyopathy questionnaire score quartile				*P*-value
		Group 1 (*n* = 228)	Group 2 (*n* = 109)	Group 3 (*n* = 545)	Group 4 (*n* = 226)	
Demographic and clinical characteristics						
Age, M (SD)	67.90 ± 14.86	70.66 ± 11.42	67.82 ± 15.46	67.20 ± 15.51	66.85 ± 15.73	0.111
Gender, *n* (%)						
Male	658 (59.33%)	121 (53.07%)	60 (54.55%)	339 (62.20%)	138 (61.06%)	0.075
Female	451 (40.67%)	107 (46.93%)	50 (45.45%)	206 (37.80%)	88 (38.94%)	
Educational level, *n* (%)						<0.001
Primary school and below	441 (39.766%)	123 (53.947%)	33 (30.000%)	193 (35.413%)	92 (40.708%)	<0.001
Middle School	148 (13.345%)	20 (8.772%)	14 (12.727%)	77 (14.128%)	37 (16.372%)	
High school/vocational school	344 (31.019%)	60 (26.316%)	45 (40.909%)	174 (31.927%)	65 (28.761%)	
University and above	176 (15.870%)	25 (10.965%)	18 (16.364%)	101 (18.532%)	32 (14.159%)	
Cardiac function classification, (%)						
Level 1	44 (3.968%)	0 (0.000%)	0 (0.000%)	0 (0.000%)	44 (19.469%)	0.023
Level 2	336 (30.298%)	25 (10.965%)	0 (0.000%)	190 (34.862%)	121 (53.540%)	
Level 3	478 (43.102%)	111 (48.684%)	66 (60.000%)	257 (47.156%)	44 (19.469%)	
Level 4	251 (22.633%)	92 (40.351%)	44 (40.000%)	98 (17.982%)	17 (7.522%)	
Number of hospitalizations due to heart failure, MED [IQR]	12.00 [8.00, 18.00]	22.00 [17.00,26.00]	8.00 [7.00, 12.00]	12.00 [8.00, 15.00]	6.00 [4.00, 8.00]	<0.001
Peripheral edema during hospitalization, *n* (%)	360 (32.46%)	146 (64.04%)	27 (24.55%)	137 (25.14%)	50 (22.12%)	<0.001
HR at discharge, M (SD)	72.63 ± 13.57	79.94 ± 16.43	66.85 ± 9.88	74.03 ± 12.20	64.68 ± 9.44	<0.001
SBP at 2 weeks post-discharge, mmHg, M (SD)	122.79 ± 17.42	103.25 ± 10.92	128.94 ± 7.93	126.52 ± 16.52	130.52 ± 13.58	<0.001
DBP at 2 weeks post-discharge, mmHg, M (SD)	70.17 ± 12.64	61.78 ± 10.40	71.81 ± 13.03	72.00 ± 12.13	73.43 ± 12.19	<0.001
SBP at 4 weeks post-discharge, mmHg, M (SD)	117.62 ± 17.52	99.81 ± 7.51	124.60 ± 9.64	121.01 ± 17.56	124.03 ± 15.79	<0.001
DBP at 4 weeks post-discharge, mmHg, M (SD)	76.45 ± 13.96	65.90 ± 10.32	76.10 ± 12.05	79.53 ± 14.25	79.85 ± 11.94	<0.001
HR at 3 months post-discharge, MED [IQR]	76.62 ± 11.92	90.08 ± 8.29	63.81 ± 5.19	79.03 ± 7.52	63.47 ± 5.37	<0.001
KCCQ1, MED [IQR]	87.00 [79.00,92.00]	81.00 [71.00,91.000]	67.00 [65.00,69.00]	87.00 [82.00,88.00]	93.00 [91.00,96.00]	<0.001
KCCQ2, MED [IQR]	85.00 [78.00,89.00]	65.00 [60.00,69.00]	78.00 [74.00,82.00]	86.00 [84.00,88.00]	94.00 [92.00,96.00]	<0.001
KCCQ3, MED [IQR]	86.00 [79.00,89.00]	60.00 [54.00,63.00]	88.00 [86.00,92.00]	86.00 [84.00,87.00]	94.00 [92.00,96.00]	<0.001
KCCQ4, MED [IQR]	84.00 [81.00,89.00]	58.00 [54.00,62.00]	88.00 [87.00,92.00]	84.00 [83.00,87.00]	94.00 [92.00,95.00]	<0.001
KCCQ5, MED [IQR]	84.00 [81.00,90.00]	58.00 [52.00,62.00]	90.00 [87.00,91.00]	84.00 [83.00,87.00]	94.00 [92.00,96.00]	<0.001
KCCQ6, MED [IQR]	84.00 [81.00,92.00]	45.00 [42.00,51.00]	94.00 [92.00,96.00]	84.00 [83.00,87.00]	95.00 [94.00,96.00]	<0.001
Complication						
Hypertension, *n* (%)	805 (72.59%)	189 (82.90%)	83 (75.46%)	370 (67.89%)	163 (72.12%)	<0.001
Coronary heart disease, *n* (%)	63 (5.68%)	21 (9.21%)	7 (6.36%)	29 (5.321%)	6 (2.66%)	0.025
Diabetes, *n* (%)	81 (7.30%)	67 (29.39%)	11 (10.00%)	1 (0.18%)	2 (0.89%)	<0.001
Stroke, *n* (%)	173 (15.60%)	76 (33.33%)	13 (11.82%)	68 (12.48%)	16 (7.08%)	<0.001
Atrial fibrillation, *n* (%)	77 (6.94%)	24 (10.53%)	2 (1.82%)	39 (7.16%)	12 (5.31%)	0.019
Chemistry						
BNP at admission, M (SD)	2,707.36 ± 2,559.79	4,367.45 ± 4,579.21	2,635.25 ± 1,354.63	2,423.99 ± 1,411.70	1,751.04 ± 1,223.28	<0.001
Percentage decrease in BNP during hospitalization, M (SD)	46.22 ± 19.67	27.40 ± 8.38	55.75 ± 17.51	48.82 ± 18.23	54.31 ± 19.93	<0.001
Serum sodium at discharge, M (SD)	137.65 ± 6.02	128.64 ± 5.25	140.04 ± 3.36	140.00 ± 3.36	139.93 ± 3.77	<0.001
Serum urea at discharge, M (SD)	9.93 ± 3.15	12.10 ± 4.42	8.97 ± 2.43	9.39 ± 2.52	9.52 ± 2.24	<0.001
BNP at discharge, M (SD)	961.70 ± 1,071.96	1,658.93 ± 1,917.22	1,043.71 ± 1,147.40	711.52 ± 359.27	821.68 ± 577.24	<0.001

HR, heart rate; SBP, systolic blood pressure; DBP, diastolic blood pressure; BNP, B-type Natriuretic Peptide.

### Association between KCCQ trajectories and cardiovascular events

Multivariable logistic regression analysis showed that KCCQ symptom trajectory groups were associated with the occurrence of MACEs. In the overall population, using the stable-intermediate group as the reference, the persistently declining group had higher odds of MACEs in the crude model (OR = 2.239, 95% CI: 1.584–3.160, *P* < 0.001), Model 1 (OR = 2.301, 95% CI: 1.611–3.288, *P* < 0.001), and Model 2 (OR = 2.242, 95% CI: 1.073–4.718, *P* = 0.032). In contrast, the gradually improving group and the stable high-score group were associated with lower odds of MACEs across the crude and adjusted models. After full adjustment, the corresponding ORs were 0.416 (95% CI: 0.184–0.843, *P* = 0.022) and 0.277 (95% CI: 0.131–0.538, *P* < 0.001), respectively. In the fully adjusted subgroup analyses, the stable high-score group remained associated with lower odds of MACEs among patients without hypertension (OR = 0.087, 95% CI: 0.005–0.491, *P* = 0.024), patients with hypertension (OR = 0.345, 95% CI: 0.154–0.714, *P* = 0.006), and patients without peripheral edema (OR = 0.290, 95% CI: 0.122–0.627, *P* = 0.003). The gradually improving group was also associated with lower odds of MACEs among patients without peripheral edema (OR = 0.313, 95% CI: 0.112–0.742, *P* = 0.014). The subgroup analyses were exploratory and should be interpreted cautiously because of multiple comparisons and relatively small sample sizes in some subgroupsDetailed results are presented in Table 4.

**Table 4:** Multivariable logistic regression and subgroup analysis of KCCQ score trajectories and cardiovascular event risk.

Predictor	Group	*N*	Case	Crude		Model 1		Model 2	
				OR (95%CI)	*P*	OR (95%CI)	*P*	OR (95%CI)	*P*
Total	Persistently Declining Group	228	80	2.239 (1.584–3.160)	<0.001	2.301 (1.611–3.288)	0.0	2.242 (1.073–4.718)	0.032
	Gradually Improving Group	110	9	0.369 (0.169–0.716)	0.006	0.353 (0.161–0.687)	0.004	0.416 (0.184–0.843)	0.022
	Stable Intermediate Group	545	106	Ref	Ref	Ref			
	Stable High-Score Group	226	12	0.232 (0.119–0.415)	<0.001	0.234 (0.120–0.418)	0.0	0.277 (0.131–0.538)	<0.001
Without hypertension	Persistently Declining Group	39	13	2.323 (1.054–4.972)	0.032	2.816 (1.226–6.382)	0.013	5.160 (0.755–38.275)	0.099
	Gradually Improving Group	27	2	0.372 (0.058–1.340)	0.193	0.306 (0.047–1.127)	0.124	0.331 (0.047–1.396)	0.181
	Stable Intermediate Group	175	31	Ref	Ref	Ref			
	Stable High-Score Group	63	2	0.152 (0.024–0.525)	0.012	0.160 (0.025–0.557)	0.014	0.087 (0.005–0.491)	0.024
With hypertension	Persistently Declining Group	189	67	2.160 (1.460–3.196)	<0.001	2.178 (1.453–3.269)	0.0	2.153 (0.942–4.973)	0.07
	Gradually Improving Group	83	7	0.362 (0.147–0.768)	0.015	0.355 (0.144–0.754)	0.013	0.423 (0.165–0.948)	0.051
	Stable Intermediate Group	370	75	Ref	Ref	Ref			
	Stable High-Score Group	163	10	0.257 (0.122–0.489)	<0.001	0.257 (0.122–0.490)	0.0	0.345 (0.154–0.714)	0.006
Without Peripheral Edema	Persistently Declining Group	82	25	1.885 (1.095–3.182)	0.019	1.991 (1.135–3.436)	0.014	2.522 (0.864–7.454)	0.091
	Gradually Improving Group	83	6	0.335 (0.127–0.738)	0.013	0.324 (0.122–0.718)	0.011	0.313 (0.112–0.742)	0.014
	Stable Intermediate Group	408	77	Ref	Ref	Ref			
	Stable High-Score Group	176	10	0.259 (0.123–0.491)	0.0	0.256 (0.122–0.487)	0.0	0.290 (0.122–0.627)	0.003
With Peripheral Edema	Persistently Declining Group	146	55	2.251 (1.335–3.857)	0.003	2.332 (1.361–4.067)	0.002	2.000 (0.625–6.612)	0.248
	Gradually Improving Group	27	3	0.466 (0.106–1.454)	0.237	0.445 (0.100–1.414)	0.216	0.697 (0.146–2.466)	0.606
	Stable Intermediate Group	137	29	Ref	Ref	Ref			
	Stable High-Score Group	50	2	0.155 (0.024–0.544)	0.013	0.157 (0.025–0.558)	0.014	0.224(0.033–0.868)	0.059

Model 1: adjusted for sex, age, and education level. Model 2 was further adjusted for NYHA functional class, diabetes mellitus, stroke, coronary artery disease, atrial fibrillation, serum sodium at discharge, number of heart failure hospitalizations, BNP at discharge, heart rate at discharge, and serum urea at discharge.

### KCCQ and trajectory ROC curves

**Figure 4:**
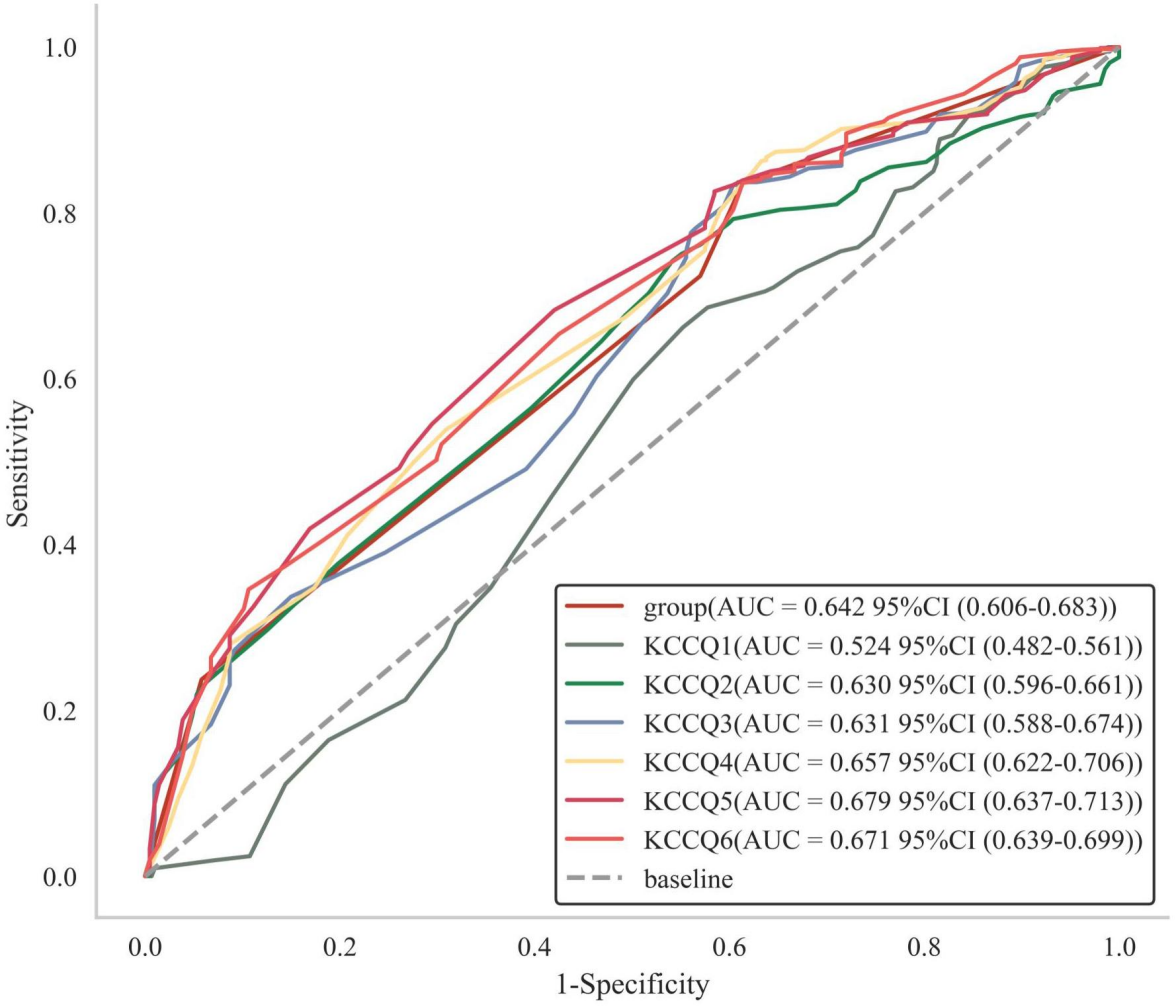
Receiver operating characteristic curves of KCCQ scores and trajectories for predicting major adverse cardiovascular events.

ROC curves were used to descriptively evaluate the discriminative performance of KCCQ scores (KCCQ1–KCCQ6) and KCCQ trajectory patterns for MACEs. These ROC analyses were unadjusted and were not externally validated; therefore, the results should be interpreted as exploratory. As shown in Figure 4, the AUC values for KCCQ1–KCCQ6 were 0.524 (95% CI: 0.482–0.561), 0.630 (95% CI: 0.596–0.661), 0.631 (95% CI: 0.588–0.674), 0.657 (95% CI: 0.622–0.706), 0.679 (95% CI: 0.637–0.713), and 0.671 (95% CI: 0.639–0.699), respectively. These values indicate modest to moderate discrimination rather than strong predictive ability. Although the AUC values tended to be higher at later follow-up time points, later KCCQ measurements may partly reflect concurrent disease severity rather than independent prospective prediction. KCCQ trajectory patterns also showed exploratory discriminative value for MACEs occurrence. Overall, these findings suggest that KCCQ scores and trajectory patterns may provide complementary prognostic information for cardiovascular risk assessment, but their predictive utility requires further validation. To further explore the association between KCCQ scores and MACEs, restricted cubic spline (RCS) models were used to assess potential nonlinear associations, and an exploratory mediation analysis was conducted to examine whether trajectory patterns may partly account for the observed associations (Figure 4).

### Restricted cubic spline analysis of KCCQ scores

**Figure 5:**
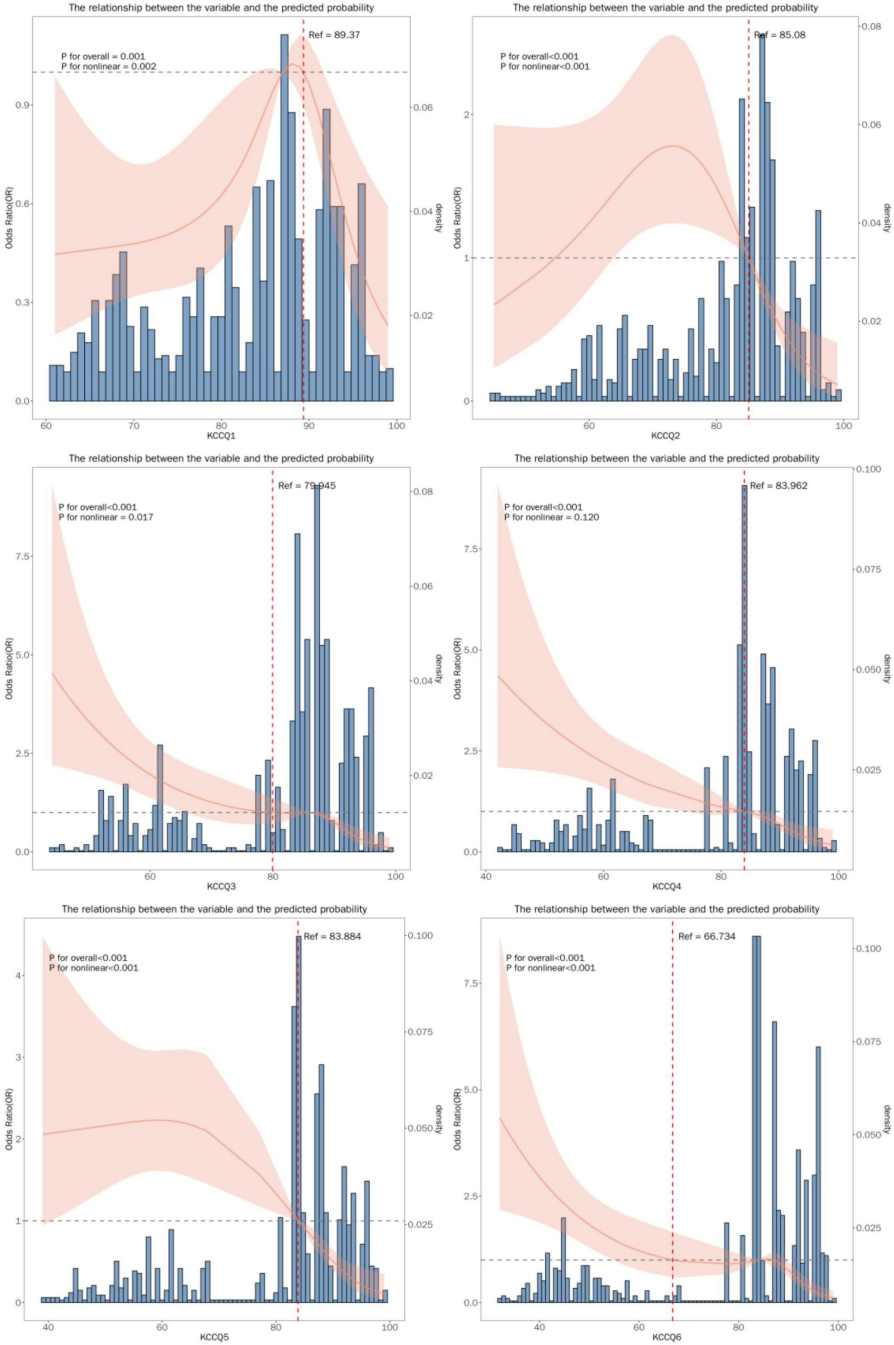
Restricted cubic spline curves showing the nonlinear association between KCCQ scores and cardiovascular event risk. KCCQ1 to KCCQ6 represent scores measured at six sequential follow-up time points. The solid line indicates the estimated hazard ratio and the shaded area shows the 95 percent confidence interval.

To complement the trajectory-based findings, restricted cubic spline analysis was used to further evaluate the association between continuous KCCQ total scores and MACEs at different time points. RCS analysis revealed that KCCQ scores at different follow-up time points were predominantly associated with cardiovascular event risk in a significant nonlinear manner. Specifically, KCCQ scores measured on day 3 post-admission (model *P* = 0.001, nonlinearity *P* = 0.002), at discharge (model *P* < 0.001, nonlinearity *P* < 0.001), at 1 month after discharge (model *P* < 0.001, nonlinearity *P* = 0.017), at 2 months after discharge (model *P* < 0.001, nonlinearity *P* = 0.012), at 3 months after discharge (model *P* < 0.001, nonlinearity *P* < 0.001), and at 6 months after discharge (model *P* < 0.001, nonlinearity *P* < 0.001) all demonstrated significant nonlinear associations. These findings indicate that the risk of cardiovascular events varied across different score ranges in a pattern that cannot be fully explained by a simple linear relationship. In contrast, while the overall model for the 2-month KCCQ score was statistically significant, its nonlinear component did not reach significance, suggesting that the relationship at this time point may be more consistent with linearity. All models were fitted using four knots. See Figure 5 for details.

### Mediation analysis

An exploratory mediation analysis was conducted to examine whether KCCQ trajectory patterns may partly account for the association between KCCQ scores and MACEs. Given the observational design, the categorical nature of trajectory membership, and the potential temporal overlap between mediator and outcome, these findings should be interpreted cautiously and regarded as hypothesis-generating.

**Figure 6:**
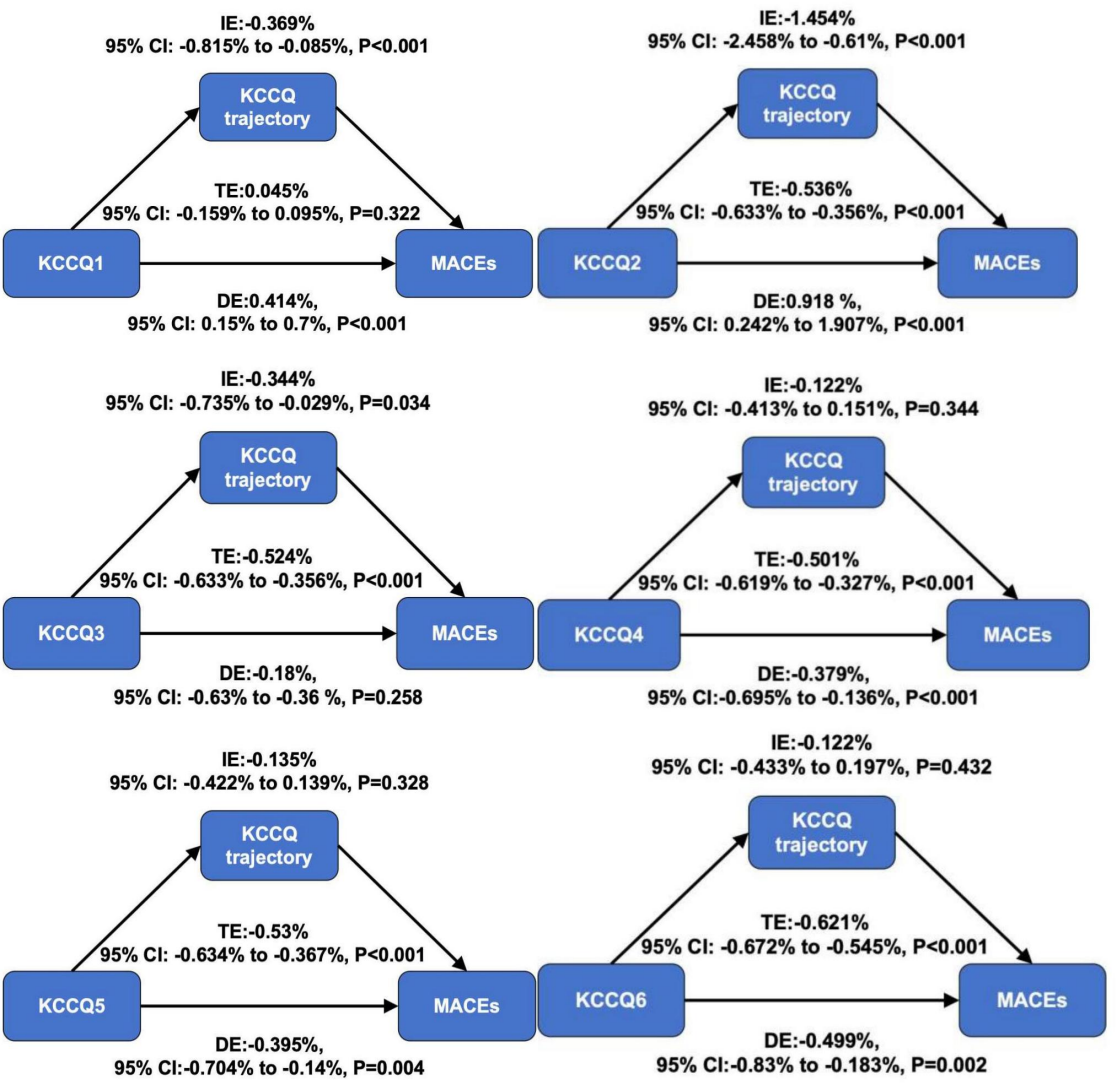
Direct and indirect effects of baseline KCCQ on cardiovascular events through symptom trajectories.

Regarding the risk of cardiovascular events, the estimated exploratory mediation proportions of different KCCQ trajectories were 65.6%, 24.4%, 25.5%, and 19.6% for KCCQ3, KCCQ4, KCCQ5, and KCCQ6, respectively. In contrast, trajectory patterns showed a masking effect for KCCQ1 and KCCQ2, attenuating or offsetting their original associations with MACEs (Figure 6). These findings suggest that KCCQ trajectories may contribute to the observed associations between KCCQ scores and cardiovascular outcomes, but they should not be interpreted as evidence of causal mediation.

The mediation analysis showed that baseline KCCQ scores were positively associated with subsequent health-related quality-of-life trajectories (*β* = 0.057, *P* < 0.001), suggesting that higher baseline KCCQ scores were associated with a greater probability of belonging to a more favorable trajectory group. In contrast, quality-of-life trajectories were negatively associated with cardiovascular events (*β* = −0.082, *P* < 0.001), indicating that more favorable trajectory patterns were associated with lower cardiovascular risk (Table 5).

**Table 5:** Direct and indirect effects of baseline KCCQ on cardiovascular events through symptom trajectories.

Path	*β*	SE	*P* val	CI [2.5%]	CI [97.5%]	sig
KCCQ trajectory∼Baseline KCCQ	0.057	0.003	0.000	0.051	0.062	Yes
Cardiovascular events∼KCCQ trajectory	−0.082	0.011	0.000	−0.104	−0.060	Yes
Total	0.002	0.001	0.133	−0.001	0.004	No
Direct	0.009	0.001	0.000	0.006	0.012	Yes
Indirect	−0.007	0.001	0.000	−0.009	−0.005	Yes

Overall effect analysis showed that the direct association between baseline KCCQ and cardiovascular events was not statistically significant (*β* = 0.002, *P* = 0.133), suggesting that baseline KCCQ alone had limited discriminative ability for MACEs in this exploratory framework. Effect decomposition showed a statistically significant indirect association through trajectory patterns (indirect effect: *β* = −0.007, 95% CI:−0.010 to −0.005, *P* < 0.001). After adjustment for trajectory group, the direct effect of baseline KCCQ became positive and statistically significant (*β* = 0.009, *P* < 0.001). However, the opposing directions of the direct and indirect associations may reflect model specification, residual confounding, or the complex relationships among baseline health status, subsequent trajectory patterns, and cardiovascular outcomes, rather than a true biological mechanism (Table 5).

Therefore, this exploratory mediation analysis should be interpreted with caution. These findings suggest that jointly considering baseline KCCQ scores and dynamic trajectory patterns may provide additional information for risk stratification, but further studies with clearer temporal ordering, more comprehensive covariate adjustment, and external validation are needed to confirm these associations.

## Discussion

In this large-scale longitudinal study, we employed a latent class trajectory model to characterize symptom trajectories assessed using the KCCQ in patients with HF during the vulnerable phase and systematically assessed their association with MACEs. Our findings revealed substantial heterogeneity in symptom evolution, with four distinct trajectories identified: persistently declining, gradually improving, stable-moderate, and stable-high. Patients in the persistently declining group exhibited the highest event risk, whereas those in the gradually improving or stable-high groups had the lowest risk. These results support the view that single time-point symptom measurements may be insufficient to fully reflect patient risk and that longitudinal symptom changes may provide additional prognostic information.

### Independent and joint effects of baseline KCCQ and symptom trajectories

Previous studies have demonstrated the prognostic value of baseline KCCQ scores in predicting cardiovascular outcomes (14, 15). Emerging research in cardiovascular medicine has emphasized the importance of baseline metrics, longitudinal trajectories, and symptom burden as complementary temporal indicators (16–18). In our study, patients with lower baseline KCCQ total scores were characterized by lower educational levels, higher NYHA class, reduced heart rate and blood pressure, and a higher prevalence of comorbidities including hypertension, stroke, coronary artery disease, diabetes, and atrial fibrillation. These patients also had more frequent prior hospitalizations, peripheral edema, elevated BNP, lower serum sodium, and higher serum urea, all of which were associated with increased MACE risk. These findings suggest that lower baseline KCCQ total scores may reflect greater clinical burden and are associated with adverse cardiovascular outcomes, consistent with findings by Kao et al. (19). Trajectory analysis further demonstrated that patients in the persistently declining group had the highest MACE risk, while those in the gradually improving or stable-high groups had markedly lower risk. Importantly, even patients with high baseline scores who exhibited a declining trajectory remained at elevated risk, aligning with observations by Lv et al. (20). Conversely, patients with low baseline scores but improving trajectories experienced significant risk reduction. These findings suggest that combining baseline status with symptom trajectories allows for a more comprehensive risk assessment: baseline scores reflect current disease burden, while trajectories capture temporal progression, providing complementary information. The trajectory-based analytic framework used in this study should be interpreted as one useful approach for characterizing dynamic changes in KCCQ total scores during the early post-discharge period, rather than as a definitive characterization of the relationship between KCCQ changes and cardiovascular outcomes. Although the identified trajectory groups were associated with MACEs, these findings should be viewed as complementary to, rather than a replacement for, single-time-point KCCQ assessments and conventional clinical risk indicators. Further studies are needed to validate these trajectory patterns in external cohorts and to determine their incremental prognostic value in different clinical settings.

Although the present study did not use Markov modeling, previous studies using serial KCCQ assessments provide an important complementary perspective. Pokharel et al. (9) reported that the most recent KCCQ score was most strongly associated with subsequent death and cardiovascular hospitalization, suggesting that current health status may capture substantial prognostic information from prior assessments. More recently, Kim et al. (21) applied a Markov-based framework to describe transitions between health states in patients with heart failure, highlighting the dynamic and bidirectional nature of health-status changes over time. In contrast, our study used group-based trajectory modeling to identify latent subgroups with distinct longitudinal patterns of KCCQ total scores during the early post-discharge period. Therefore, our findings should be interpreted as complementary to, rather than a replacement for, the Markov-based perspective. While Markov-based approaches emphasize transitions between current health states and updated risk assessment, trajectory-based modeling may help characterize clinically interpretable symptom-evolution patterns at the population level. Further studies are needed to compare these approaches directly and to evaluate their incremental prognostic value in different heart failure populations.

### Independent predictive value of KCCQ trajectories and stratified analysis

Analysis of baseline characteristics and KCCQ scores confirmed that both exerted significant influence on cardiovascular outcomes, with stratified analysis highlighting the prognostic importance of trajectories. Multivariable logistic regression showed that even after adjusting for sex, age, serum sodium, BNP, and cardiac function, symptom trajectories remained associated with cardiovascular events. This finding suggests that dynamic symptom changes may provide information beyond a single baseline measurement, consistent with prior studies. Huang et al. noted that symptom burden is a key determinant of post-discharge outcomes (22), and multiple studies have confirmed the relationship between KCCQ scores and prognosis across different settings, including acute decompensated HF hospitalization, one-week post-discharge, and outpatient follow-up (9, 23–25). Our stratified analysis revealed that symptom trajectories appeared to have greater prognostic relevance in patients lacking typical clinical risk factors. Among patients without hypertension, the persistently declining group had significantly elevated event risk, whereas in hypertensive patients, the trend did not reach significance, suggesting that hypertension may partially obscure the predictive utility of trajectories. Similarly, in patients without peripheral edema, the persistently declining trajectory was associated with significantly higher risk, but not in those with edema. This aligns with Liu et al.'s findings on the prognostic significance of congestion (26), indicating that strong baseline predictors may attenuate the independent effect of KCCQ trajectories. These results emphasize that clinical interpretation of trajectories should consider baseline characteristics, particularly in patients without overt risk indicators.

### Mediating role of baseline KCCQ via symptom trajectories

Mediation analysis provided further insight into the relationship between baseline KCCQ and cardiovascular events. Baseline scores influenced outcomes indirectly through symptom trajectories; however, within the same trajectory, higher baseline KCCQ levels were paradoxically associated with increased risk. This suggests the existence of a hidden high-risk subgroup—patients with mild baseline symptoms whose trajectories failed to improve or worsened over time. Reliance on single baseline assessments may underestimate risk, leading to insufficient follow-up or intervention. Decomposing the total effect revealed a bidirectional influence: trajectory improvement conferred a protective effect, while controlling for trajectory showed elevated risk for those with declining or stagnating trajectories, resulting in a nonsignificant overall effect. Thus, integrating baseline status with dynamic trajectories provides a more comprehensive assessment, enhancing risk stratification and enabling individualized management.

### Clinical implications

Clinically, these findings advocate for a shift from static to dynamic risk assessment. Corbin and Strauss' chronic illness trajectory framework emphasizes that disease processes are multidimensional and evolving, necessitating adaptive care strategies (27). Patients with persistently declining trajectories or hidden high-risk profiles require intensified post-discharge monitoring, optimized pharmacotherapy, and enhanced lifestyle and self-management support to prevent further deterioration (28, 29). Conversely, patients with stable or improving trajectories may be managed with standard therapy while avoiding unnecessary interventions, allowing for more efficient allocation of healthcare resources.

### Strengths and limitations

This study has several strengths. First, leveraging a large cohort with long-term follow-up allowed for a comprehensive characterization of the dynamic evolution of HF symptoms. Second, repeated KCCQ assessments during follow-up combined with outcome events provided a robust basis for examining the relationship between symptom trajectories and clinical outcomes. In addition, by applying trajectory analysis to repeated symptom assessment during the vulnerable phase, this study provides additional evidence on post-discharge symptom evolution and its association with cardiovascular events in a Chinese HF cohort. However, several limitations should be acknowledged. As an observational study, the lack of randomization and interventional measures precludes definitive causal inference, and residual unmeasured confounding cannot be entirely excluded. Although missing data were handled using multiple imputation, the possibility of informative missingness in patient-reported KCCQ assessments could not be completely ruled out. In addition, because repeated KCCQ measurements are naturally correlated over time, the trajectory groups derived using GBTM may partly reflect serial dependence in KCCQ values. Therefore, some information captured by trajectory groups may also be represented by the most recent KCCQ score or by simpler Markov-type models, and the present findings should be interpreted within the assumptions of the selected trajectory-modeling framework. Second, this was a single-center study conducted in a Class III Grade A hospital in China, which may limit the generalizability of the findings. The identified KCCQ trajectory patterns may be influenced by local patient characteristics, clinical practice patterns, and healthcare system context; therefore, external validation in multicenter cohorts and other healthcare settings is needed. The follow-up period was relatively limited, potentially underestimating late events or long-term trends, highlighting the need for extended observation to strengthen the robustness of findings. Future studies should aim to expand sample size, incorporate additional clinical and biological markers, explore interactions among multiple trajectories, and develop intuitive visual predictive models to enhance clinical applicability.

## Conclusions

Using a latent class trajectory model, this study identified distinct KCCQ symptom trajectory patterns during the early post-discharge period after HF hospitalization. These trajectory patterns were associated with subsequent MACEs. Compared with patients with stable-moderate symptoms, those with persistently declining trajectories had higher odds of MACEs, whereas those with gradually improving or stable high-score trajectories showed more favorable outcomes. These findings suggest that dynamic KCCQ trajectory patterns may provide complementary prognostic information beyond single-time-point assessment. Further multicenter studies with external validation are needed to confirm their clinical utility and to determine the optimal timing of KCCQ assessments for risk stratification.

## Data Availability

The raw data supporting the conclusions of this article will be made available by the authors, without undue reservation.
