# Supplementary material for: Trajectories of symptom scores and risk of cardiovascular events in the vulnerable phase of heart failure: an analysis using a latent class trajectory model
Source: Front Cardiovasc Med. 2026 Jul 20;13:1841870. doi: 10.3389/fcvm.2026.1841870 (PMC13429835; doi:10.3389/fcvm.2026.1841870)
Supplement: Supplementary file 1 [file Datasheet1.docx]

Supplementary Table S1. Sensitivity analysis of polynomial specifications in the four-group trajectory model

| Degree | BIC | AIC | Entropy |
| --- | --- | --- | --- |
| 1 | 40015.70 | 39940.53 | 1.0135 |
| 2 | 39645.12 | 39549.91 | 1.0048 |
| 3 | 41613.45 | 41498.19 | 1.0027 |

Notes: Degree 1, linear specification; Degree 2, quadratic specification; Degree 3, cubic specification. The quadratic model showed the lowest AIC and BIC values and was selected as the final trajectory specification.

**Supplementary Table S2. Group proportions and average posterior probabilities in the final four-group quadratic model**

| **Value** | **Group1** | **Group2** | **Group3** | **Group4** |
| --- | --- | --- | --- | --- |
| group_rate | 0.206 | 0.098 | 0.492 | 0.204 |
| AvePP | 1 | 0.998 | 0.996 | 0.998 |

Notes: All average posterior probabilities exceeded 0.70, indicating adequate classification accuracy.
